# Factors associated with suboptimal adherence to antiretroviral therapy in Asia

**DOI:** 10.7448/IAS.17.1.18911

**Published:** 2014-05-16

**Authors:** Awachana Jiamsakul, Nagalingeswaran Kumarasamy, Rossana Ditangco, Patrick CK Li, Praphan Phanuphak, Thira Sirisanthana, Somnuek Sungkanuparph, Pacharee Kantipong, Christopher KC Lee, Mahiran Mustafa, Tuti Merati, Adeeba Kamarulzaman, Thida Singtoroj, Matthew Law, on behalf of the TREAT Asia Studies to Evaluate Resistance Monitoring Study (TASER-M)

**Affiliations:** 1 The Kirby Institute, UNSW Australia, Sydney, Australia;; 2 YRGCARE Medical Centre, Chennai, India; 3 Research Institute for Tropical Medicine, Manila, Philippines; 4 Department of Medicine, Queen Elizabeth Hospital, Hong Kong, China; 5 Faculty of Medicine, Chulalongkorn University and HIV-NAT/Thai Red Cross AIDS Research Centre, Bangkok, Thailand; 6 Research Institute for Health Sciences, Chiang Mai University, Chiang Mai, Thailand; 7 Faculty of Medicine Ramathibodi Hospital, Mahidol University, Bangkok, Thailand; 8 Chiangrai Prachanukroh Hospital, Chiang Rai, Thailand; 9 Hospital Sungai Buloh, Sungai Buloh, Malaysia; 10 Hospital Raja Perempuan Zainab II, Kota Bharu, Malaysia; 11 Udayana University, Sanglah Hospital, Bali, Indonesia; 12 University of Malaya Medical Center, Kuala Lumpur, Malaysia; 13 TREAT Asia, amfAR – The Foundation for AIDS Research, Bangkok, Thailand

**Keywords:** HIV, adherence, Asia, resource-limited, visual analogue scale

## Abstract

**Introduction:**

Adherence to antiretroviral therapy (ART) plays an important role in treatment outcomes. It is crucial to identify factors influencing adherence in order to optimize treatment responses. The aim of this study was to assess the rates of, and factors associated with, suboptimal adherence (SubAdh) in the first 24 months of ART in an Asian HIV cohort.

**Methods:**

As part of a prospective resistance monitoring study, the TREAT Asia Studies to Evaluate Resistance Monitoring Study (TASER-M) collected patients’ adherence based on the World Health Organization-validated Adherence Visual Analogue Scale. SubAdh was defined in two ways: (i) <100% and (ii) <95%. Follow-up time started from ART initiation and was censored at 24 months, loss to follow-up, death, treatment switch, or treatment cessation for >14 days. Time was divided into four intervals: 0–6, 6–12, 12–18 and 18–24 months. Factors associated with SubAdh were analysed using generalized estimating equations.

**Results:**

Out of 1316 patients, 32% ever reported <100% adherence and 17% ever reported <95%. Defining the outcome as SubAdh <100%, the rates of SubAdh for the four time intervals were 26%, 17%, 12% and 10%. Sites with an average of >2 assessments per patient per year had an odds ratio (OR)=0.7 (95% confidence interval (CI) (0.55 to 0.90), *p*=0.006), compared to sites with ≤2 assessments per patient per year. Compared to heterosexual exposure, SubAdh was higher in injecting drug users (IDUs) (OR=1.92, 95% CI (1.23 to 3.00), *p*=0.004) and lower in homosexual exposure (OR=0.52, 95% CI (0.38 to 0.71), *p*<0.001). Patients taking a nucleoside transcriptase inhibitor and protease inhibitor (NRTI+PI) combination were less likely to report adherence <100% (OR=0.36, 95% CI (0.20 to 0.67), *p*=0.001) compared to patients taking an NRTI and non-nucleoside transcriptase inhibitor (NRTI+NNRTI) combination. SubAdh decreased with increasing time on ART (all *p*<0.001). Similar associations were found with adherence <95% as the outcome.

**Conclusions:**

We found that SubAdh, defined as either <100% and <95%, was associated with mode of HIV exposure, ART regimen, time on ART and frequency of adherence measurement. The more frequently sites assessed patients, the lower the SubAdh, possibly reflecting site resourcing for patient counselling. Although social desirability bias could not be excluded, a greater emphasis on more frequent adherence counselling immediately following ART initiation and through the first six months may be valuable in promoting treatment and programme retention.

## Introduction

Adherence to antiretroviral therapy (ART) plays a critical role in optimizing HIV treatment outcomes. Poor or suboptimal adherence (SubAdh) has been associated with virological failure, poor CD4 response as well as an increased risk of developing drug resistance [1–3]. To maximize the potential long-term success of ART, it is important to identify risk factors contributing to SubAdh.

Previous studies have shown that factors such as developing side effects, having been in clinical stage B/C, being in a younger age group and using alcohol were significant contributors to SubAdh [4, 5]. In resource-limited settings, social factors such as the cost of antiretrovirals and/or healthcare, non-HIV disclosure, travel time and stigma have been some of the reasons related to the failure to adhere to ART [4, 6, 7]. In such settings, access to second-line ART may be restricted, and some countries may only recommend switching once patients have been assured to maintain good adherence [8].

There are many methods available to monitor ART adherence. Self-reported assessments have been shown to be an appropriate tool for measuring adherence in resource-limited settings due to their ease of use and low cost. Studies suggest that although self-reported methods tend to overestimate actual adherence, they comprise a valid tool that can be used to measure levels of antiretroviral adherence, especially in low-income countries [9–11]. Moreover, a previous study investigating drug resistance mutations in our HIV cohort found that there was an association between self-reported adherence and virological failure [12].

The objectives of this study were to investigate the rates of SubAdh and the effects of demographic and clinical parameters on the association with SubAdh in the first 24 months after ART initiation, based on the 30-day self-reported adherence assessments in an Asian adult HIV cohort.

## Methods

### Study population

**Figure 1:**
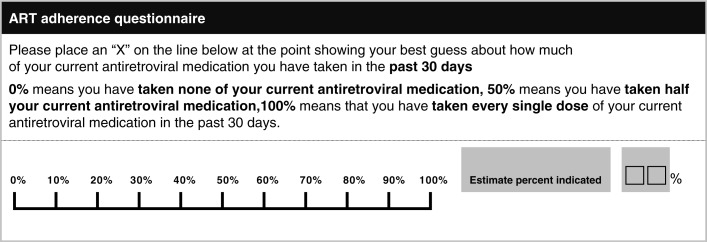
TASER-M adherence visual analogue scale.

The Therapeutics, Research, Education and AIDS Training in Asia (TREAT Asia) Studies to Evaluate Resistance Monitoring (TASER-M), began recruitment in 2007 and included 12 clinical sites from Thailand, Hong Kong, Malaysia, the Philippines and Indonesia. The primary goal of TASER-M was to monitor HIV drug resistance mutations in Asia. Patients were enrolled as either treatment naïve (those initiating ART) or treatment experienced (those switching to second-line therapy) [13]. TASER-M study sites collected ART adherence data based on the World Health Organization (WHO)-endorsed [14] 30-day self-reported adherence visual analogue scale (VAS) (Figure 1). Patients were included in the analysis if they were enrolled as treatment naïve and initiated ART between 2007 and 2011, with at least one adherence assessment prior to the analysis censoring date. Follow-up time started from ART initiation and censored at 24 months after ART initiation, death, loss to follow-up, treatment cessation of more than 14 days, or treatment switch, the latter defined by a change of at least two drugs or one drug class from the initial treatment combination, whichever occurred first. The analysis was censored at treatment cessation or treatment switch to ensure that the adherence levels were those that corresponded to first-line ART adherence.

Data transfers from clinical sites were aggregated at The Kirby Institute, University of New South Wales, Australia, at six-monthly intervals in March and September. Ethics approvals were obtained from the University of New South Wales Ethics Committee and institutional review boards at the participating clinical sites and coordinating centre (TREAT Asia/The Foundation for AIDS Research (amfAR), Bangkok, Thailand). Written informed consent was obtained from participants prior to enrolment.

### Definition

SubAdh was defined in two ways: (i) adherence <100% and (ii) adherence <95%, in the past 30 days according to the VAS. The adherence percentage indicated in the two boxes to the right of the linear scale (Figure 1) was used in the analysis. Cut-off point (i) was chosen to distinguish those who did not fully adhere to ART compared to those who achieved 100% adherence, and cut-off point (ii) was chosen based on the traditional cut-off point found in previous literature to be associated with virologic failure [14].

Follow-up time since start of ART was divided into four 6-monthly intervals: 0 to 6, 6 to 12, 12 to 18 and 18 to 24 months. If multiple adherence assessments were recorded in any given time interval, the lowest adherence level in that interval was used. Intervals missing an adherence record were kept as missing.

We created a variable to represent site-level resources [15, 16] for adherence management that summarized the frequency of adherence assessments for individual sites. Each site was assigned a number that measured the average frequency of assessments for the entire follow-up duration. All patients belonging to the same site were then classified according to their site's frequency level, rather than their individual adherence assessments. The frequency was obtained by averaging the number of adherence records per individual patient per year. For each site, the median of the average number of VAS results for all patients was then determined. Sites were then categorized into groups either above or below the overall median frequency for all sites.

### Statistical analysis

Factors associated with SubAdh were analysed using generalized estimation equations (GEEs) with binomial distribution, logit link function, exchangeable correlation structure and robust standard errors, to account for repeated measures in different patients (GEE-logit). The exchangeable correlation matrix was chosen due to its simplicity, whereby only one correlation coefficient has to be estimated.

Variables included in the univariate analysis were age, sex, exposure category, CD4, initial ART category, hepatitis B and C co-infection, AIDS diagnosis, adverse events, site adherence frequency and time on ART. We did not adjust for viral load in the regression models due to concerns over causality between viral load and adherence. Variables significant in the univariate analysis at the 10% level were included in the multivariate model by a backward stepwise selection process. Factors were considered significant and adjusted for in the final multivariate model if *p*<0.05. CD4, AIDS diagnosis and adverse events were modelled as time-updated variables, lagged forward to the next time interval. For CD4 cell count, if more than one measurement was available during each of the six-month intervals, the average of the measurements was used for that interval. For AIDS diagnosis, once a patient was diagnosed as having an AIDS-defining illness, that patient would be coded in this category for the remaining follow-up time. Adverse events were coded as ever having events of grades 3 (severe), 4 (life-threatening) or 5 (causing death) in each of the time intervals. Multiple sensitivity analyses were performed:

- The group of patients who were considered lost as of March 2012 (i.e. patients who had not been seen between March 2011 and March 2012) had their follow-up time extended to 24 months. Missing adherence, CD4, AIDS diagnosis and adverse events for these lost patients were imputed using last observation carried forward (LOCF) methods;
- All patient follow-up time was extended to 24 months regardless of their status. Any time interval without an adherence assessment, CD4, AIDS diagnosis and adverse events was imputed using LOCF. Adherence was also filled backwards to earlier time intervals, if missing, using the first assessment. As such, a patient with only one adherence assessment would have that adherence for the entire 24-month duration;
- The site adherence assessment variable was removed, and the model was adjusted by country income status [17];
- The site adherence assessment variable was removed, and the model was adjusted by individual countries; and
- The analysis was performed using a GEE with Poisson distribution (GEE-Poisson), and the incidence rate ratios (IRRs) were reported.

For all sensitivity analyses, SubAdh was defined as VAS self-reports <100%.

All data management and statistical analyses were performed using SAS software version 9.2 (SAS Institute Inc., Cary, NC, USA) and STATA software version 12.1 (STATA Corp., College Station, TX, USA).

## Results

A total of 1316 patients from 11 sites who were enrolled in TASER-M and initiated ART with at least one adherence assessment available within the first 24 months of ART, prior to the censoring date, were included. One site was excluded as adherence information was not collected. Table 1 shows the descriptive characteristics of patients at treatment initiation. The median age was 36 years (interquartile range (IQR): 31 to 43). The majority of patients were male, with heterosexual contact as the main mode of HIV exposure. Median pre-treatment CD4 was 106 cells/µL (IQR: 35 to 206). Patients were primarily started on a nucleoside transcriptase inhibitor and non-nucleoside transcriptase inhibitor (NRTI+NNRTI) combination (93%), with the majority having no hepatitis B or C co-infection. Prior AIDS diagnosis was also absent in 63% of patients. The number of patients with 100% adherence consistently reported throughout the entire analysis time was 890/1316 (68%).

**Table 1:** Demographics

	Total (%)	All adherence 100% (%)	At least 1 adherence <100% (%)
			
	*n*=1316 (100)	*n*=890 (68)	*n*=426 (32)
Age, years			
≤30	321 (24)	228 (26)	93 (22)
31–40	554 (42)	352 (40)	202 (47)
41–50	316 (24)	213 (24)	103 (24)
51+	125 (10)	97 (11)	28 (7)
Sex			
Male	879 (67)	597 (67)	282 (66)
Female	437 (33)	293 (33)	144 (34)
HIV exposure category			
Heterosexual contact	911 (69)	601 (68)	310 (73)
Homosexual contact	273 (21)	211 (24)	62 (15)
Injecting drug use	72 (5)	33 (4)	39 (9)
Other or unknown	60 (5)	45 (5)	15 (4)
Pre-treatment CD4, cells/μL			
≤50	422 (32)	291 (33)	131 (31)
51–100	200 (15)	130 (15)	70 (16)
101–200	322 (24)	204 (23)	118 (28)
201+	337 (26)	240 (27)	97 (23)
Missing	35 (3)	25 (3)	10 (2)
Initial ART regimen			
NRTI+NNRTI	1219 (93)	807 (91)	412 (97)
NRTI+ PI	76 (6)	64 (7)	12 (3)
NRTI only	21 (2)	19 (2)	2 (0)
Hepatitis B co-infection			
Negative	1030 (78)	696 (78)	334 (78)
Positive	128 (10)	89 (10)	39 (9)
Not tested	158 (12)	105 (12)	53 (12)
Hepatitis C co-infection			
Negative	950 (72)	646 (73)	304 (71)
Positive	90 (7)	49 (6)	41 (10)
Not tested	276 (21)	195 (22)	81 (19)
Previous AIDS			
No	826 (63)	549 (62)	277 (65)
Yes	490 (37)	341 (38)	149 (35)

Within six months prior to ART initiation, the median viral load was 100,000 copies/mL (IQR: 36874.50 to 230,000). Seven hundred and eighteen patients (55%) contributed a complete 24-month follow-up time to the analysis, 137 (10%) switched treatment in the first 24 months, 21 (2%) ceased treatment for more than 14 days and 440 (33%) had follow-up times of less than 24 months due to death, loss to follow-up or being newly recruited. The median frequency of adherence measurements for sites ranged from one to six per patient per year. Five sites had frequency >2 per patient per year, and six sites were ≤2 per patient per year.

### Suboptimal adherence <100%

**Figure 2:**
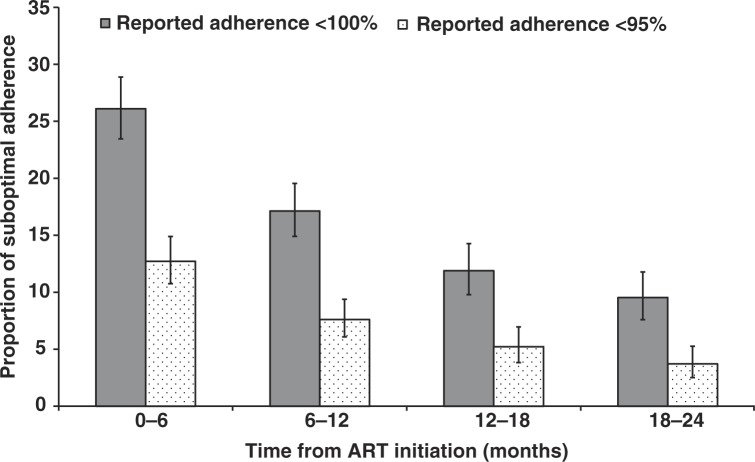
Proportion of patients reporting suboptimal adherence by visual analogue scale, with 95% confidence intervals.

The total numbers of patients with adherence assessments by time intervals were 1046 (0 to 6 months), 1051 (6 to 12 months), 841 (12 to 18 months) and 807 (18 to 24 months). The proportions of patients with self-reported adherence <100% in each of these intervals were 26%, 17%, 12% and 10% (Figure 2), with 32% of all patients reporting <100% at least once during follow-up. The first section of Table 2 shows the univariate and multivariate GEE analyses with SubAdh <100% as the outcome. Factors significant in the univariate analyses at alpha=0.10 were site adherence assessments (*p*=0.014), exposure category (*p*<0.001), CD4 (*p*<0.001), initial ART category (*p*<0.001), hepatitis C co-infection (*p*=0.012), adverse events (*p*=0.090) and time on ART (*p*<0.001). The four covariates remaining significant in the multivariate model were site adherence assessments, exposure category, initial ART and time. After adjusting for the significant covariates, sites with average adherence measurements of >2 times per patient year had a 30% reduced odds for SubAdh <100% (odds ratio (OR)=0.7, 95% confidence interval (CI) (0.55 to 0.90), *p*=0.006). Homosexual mode of exposure was associated with an almost 50% reduction (OR=0.52, 95% CI (0.38 to 0.71), *p*<0.001), while injecting drug users (IDUs) were almost twice as likely to report <100% adherence compared to the heterosexual exposure group (OR=1.92, 95% CI (1.23 to 3.00), *p*=0.004). In terms of initial ART, patients who were put on NRTI+protease inhibitor (PI) combinations were less likely to report SubAdh (OR=0.36, 95% CI (0.20 to 0.67), *p*=0.001). Increasing time on ART was also associated with decreasing odds of SubAdh (OR=0.59, 0.40 and 0.35, all *p*<0.001) for time intervals 6 to 12, 12 to 18 and 18 to 24 months, respectively, compared to the initial six months. There were no significant differences by age, sex, CD4, hepatitis B and C co-infection, AIDS diagnosis and adverse events, after adjusting for the significant factors in the final model.

**Table 2:** Factors associated with adherence <100% and <95%

	Adherence <100%						Adherence <95%					
		
	Univariate			Multivariate			Univariate			Multivariate		
				
	OR	95% CI	*p*	OR	95% CI	*p*	OR	95% CI	*p*	OR	95% CI	*p*
Site adherence assessments per patient per year												
≤2	1.00			1.00			1.00			1.00		
>2	0.75	(0.60 to 0.94)	0.014	**0.70**	**(0.55 to 0.90)**	**0.006**	0.40	(0.30 to 0.53)	<0.001	**0.39**	**(0.28 to 0.55)**	**<0.001**
Age, years			0.121			0.148			0.647			0.835
≤30	1.00			1.00			1.00			1.00		
31–40	1.16	(0.88 to 1.54)	0.292	1.05	(0.79 to 1.41)	0.724	1.16	(0.81 to 1.68)	0.418	1.08	(0.75 to 1.56)	0.685
41–50	0.98	(0.72 to 1.35)	0.920	0.92	(0.66 to 1.28)	0.614	1.08	(0.71 to 1.63)	0.720	1.08	(0.72 to 1.64)	0.700
51+	0.69	(0.43 to 1.12)	0.135	0.63	(0.39 to 1.02)	0.062	0.83	(0.45 to 1.54)	0.555	0.84	(0.45 to 1.55)	0.574
Sex												
Male	1.00			1.00			1.00			1.00		
Female	0.98	(0.78 to 1.23)	0.864	0.94	(0.73 to 1.21)	0.616	0.84	(0.62 to 1.14)	0.267	0.88	(0.63 to 1.24)	0.477
HIV exposure category			<0.001			**<0.001**			<0.001			**<0.001**
Heterosexual contact	1.00			1.00			1.00			1.00		
Homosexual contact	0.56	(0.42 to 0.75)	<0.001	**0.52**	**(0.38 to 0.71)**	**<0.001**	0.53	(0.34 to 0.82)	0.004	**0.43**	**(0.27 to 0.69)**	**<0.001**
Injecting drug use	2.67	(1.72 to 4.14)	<0.001	**1.92**	**(1.23 to 3.00)**	**0.004**	5.26	(3.34 to 8.29)	<0.001	**2.79**	**(1.72 to 4.52)**	**<0.001**
Other or unknown	0.62	(0.36 to 1.08)	0.091	0.58	(0.32 to 1.02)	0.060	0.67	(0.30 to 1.46)	0.310	0.53	(0.24 to 1.19)	0.125
CD4, cells/µL			<0.001			0.798			<0.001			0.459
≤50	1.00			1.00			1.00			1.00		
51–100	0.88	(0.65 to 1.19)	0.408	1.15	(0.82 to 1.59)	0.417	0.62	(0.40 to 0.96)	0.031	0.74	(0.46 to 1.21)	0.234
101–200	0.70	(0.54 to 0.91)	0.009	1.15	(0.85 to 1.56)	0.373	0.54	(0.37 to 0.78)	0.001	0.77	(0.50 to 1.20)	0.252
201+	0.48	(0.38 to 0.61)	<0.001	1.13	(0.83 to 1.53)	0.431	0.39	(0.28 to 0.54)	<0.001	0.72	(0.47 to 1.11)	0.141
Missing	0.59	(0.45 to 0.76)	<0.001	1.35	(0.96 to 1.90)	0.082	0.47	(0.33 to 0.68)	<0.001	0.79	(0.48 to 1.32)	0.369
Initial ART regimen			<0.001			**0.003**			0.004			**0.005**
NRTI+NNRTI	1.00			1.00			1.00			1.00		
NRTI+PI	0.32	(0.18 to 0.57)	<0.001	**0.36**	**(0.20 to 0.67)**	**0.001**	0.10	(0.02 to 0.38)	0.001	**0.10**	**(0.03 to 0.41)**	**0.001**
NRTI only	0.34	(0.07 to 1.52)	0.157	0.39	(0.08 to 1.87)	0.240	0.81	(0.18 to 3.68)	0.785	0.79	(0.16 to 3.96)	0.773
Hepatitis B co-infection			0.812			0.966			0.357			0.379
Negative	1.00			1.00			1.00			1.00		
Positive	0.96	(0.66 to 1.39)	0.812	0.99	(0.67 to 1.47)	0.966	0.77	(0.45 to 1.33)	0.357	0.77	(0.43 to 1.38)	0.379
Not tested	1.16	(0.83 to 1.63)	0.386	0.98	(0.71 to 1.35)	0.883	1.75	(1.18 to 2.58)	0.005	1.28	(0.87 to 1.87)	0.208
Hepatitis C co-infection			0.012			0.921			<0.001			0.970
Negative	1.00			1.00			1.00			1.00		
Positive	1.64	(1.12 to 2.42)	0.012	0.98	(0.62 to 1.55)	0.921	2.41	(1.57 to 3.70)	<0.001	0.99	(0.58 to 1.69)	0.970
Not tested	0.88	(0.67 to 1.17)	0.378	0.77	(0.58 to 1.03)	0.079	1.00	(0.69 to 1.45)	0.982	0.73	(0.50 to 1.06)	0.099
AIDS diagnosis												
No	1.00			1.00			1.00			1.00		
Yes	0.85	(0.68 to 1.07)	0.168	0.82	(0.65 to 1.04)	0.099	0.97	(0.73 to 1.30)	0.855	1.08	(0.79 to 1.47)	0.633
Adverse events grade ≥3												
No	1.00			1.00			1.00			1.00		
Yes	0.67	(0.42 to 1.06)	0.090	0.82	(0.52 to 1.28)	0.376	0.50	(0.21 to 1.16)	0.106	0.74	(0.31 to 1.80)	0.510
Time on ART, months			<0.001			**<0.001**			<0.001			**<0.001**
0–6	1.00			1.00			1.00			1.00		
6–12	0.59	(0.49 to 0.70)	<0.001	**0.59**	**(0.49 to 0.70)**	**<0.001**	0.58	(0.44 to 0.75)	<0.001	**0.55**	**(0.42 to 0.73)**	**<0.001**
12–18	0.40	(0.33 to 0.50)	<0.001	**0.40**	**(0.33 to 0.50)**	**<0.001**	0.41	(0.30 to 0.56)	<0.001	**0.41**	**(0.29 to 0.57)**	**<0.001**
18–24	0.34	(0.27 to 0.43)	<0.001	**0.35**	**(0.27 to 0.44)**	**<0.001**	0.32	(0.23 to 0.45)	<0.001	**0.30**	**(0.21 to 0.44)**	**<0.001**

Note: *CD4*, *AIDS diagnosis* and *adverse events* are time-updated variables.

*P*-values for test for heterogeneity excluded missing or not-tested values.

*P*-values in bold represent significant covariates in the final model.

Non-significant factors were presented in the multivariate model adjusted for significant predictors.

### Suboptimal adherence <95%

The second section of Table 2 shows the GEE results when the outcome of interest was defined as adherence of <95%. The corresponding proportions for this SubAdh for the four time intervals were 13%, 8%, 5% and 4% (Figure 2). Out of 1316 patients, 17% had at least one adherence measurement <95%. In the multivariate analysis, sites with average adherence measurements >2 times per patient per year now had a 61% reduced odds (OR=0.39, 95% CI (0.28 to 0.55), *p*<0.001) for SubAdh <95%. Those with homosexual HIV exposure were less likely to report SubAdh (OR=0.43, 95% CI (0.27 to 0.69), *p*<0.001), while IDUs had more than double the odds (OR=2.79, 95% CI (1.72 to 4.52), *p*<0.001), compared to the heterosexual exposure group. Patients who were prescribed NRTI+PI for their initial regimens showed decreased odds for SubAdh (OR=0.10, 95% CI (0.03 to 0.41), *p*=0.001) compared to the NRTI+NNRTI group. Time on ART also showed similar effects to adherence <100% (OR=0.55, 0.41 and 0.30, all *p*<0.001).

### Sensitivity analyses

Table 2 shows that adherence can be affected by clinical sites and duration of ART. It could be argued that the effects of a decreasing trend of SubAdh with a longer time from ART initiation, in other words improving adherence as time increases, could be due to the lost-to-follow-up cases. We therefore performed two sensitivity analyses to further investigate this claim. In sensitivity analysis (i), where lost patients had their follow-up time extended to 24 months and SubAdh was defined only as <100%, time on ART remained significant with decreasing ORs when compared to the first six months (6 to 12 months: OR=0.61, 95% CI (0.52 to 0.72); 12 to 18 months: OR=0.46, 95% CI (0.38 to 0.56); and 18 to 24 months: OR=0.41, 95% CI (0.34 to 0.50), all *p*<0.001). All other covariates that were significant in Table 2 were also significant in this sensitivity analysis with similar ORs and *p*-values. The same can be said for sensitivity analysis (ii), where all patients had their follow-up time extended to 24 months regardless of their follow-up status. The results were somewhat attenuated with time (6 to 12 months: OR=0.70, 95% CI (0.62 to 0.79); 12 to 18 months: OR=0.59, 95% CI (0.52 to 0.68); and 18 to 24 months: OR=0.56, 95% CI (0.48 to 0.64), all *p*<0.001). This suggested that the improvement in adherence as time on ART increases was not entirely due to loss to follow-up and missing data.

To investigate the effect of sites, we conducted sensitivity analyses (iii) and (iv), where the site adherence assessment variable was replaced by country income status and individual countries, respectively. In sensitivity analysis (iii), after controlling for exposure category, initial regimen and time on ART, the OR for having <100% adherence for sites in lower-middle-income countries was 0.71 compared to those from high-+upper-middle-income countries (95% CI (0.44 to 1.15), *p*=0.167). This indicates that there was no significant difference in adherence between the lower-middle countries and high+upper-middle countries. On the other hand, sensitivity analysis (iv) suggested that when categorizing by individual countries, the adherence effects for each country were not all the same (*p*=0.002, individual ORs not reported to allow for anonymity among sites). The significance and ORs of the other adjusted covariates in these two sensitivity models were similar to those in Table 2, where the site adherence assessment variable was used. Therefore, the use of the site adherence assessment took into account potential variation in adherence management practices without significantly altering the effects of other predictors in the model.

In sensitivity analysis (v), we re-analysed the data using a GEE-Poisson model, rather than the GEE-logit model reported in Table 2, as the OR is considered to be an approximation of the risk ratio. In the multivariate model, the same variables that were significant in the GEE-logit model were also found to be significant in the GEE-Poisson model, with similar effect sizes and significance. With the same reference categories used in Table 2, the effects for the significant covariates in the GEE-Poisson model were: sites with >2 adherence assessments per patient per year: IRR=0.77, 95% CI (0.63 to 0.93), *p*=0.006; homosexual HIV exposure: IRR=0.59, 95% CI (0.45 to 0.76), *p*<0.001; IDUs: IRR=1.52, 95% CI (1.14 to 2.01), *p*=0.004; NRTI+PI initial regimen: IRR=0.42, 95% CI (0.24 to 0.73), *p*=0.002; time on ART 6 to 12 months: IRR=0.66, 95% CI (0.58 to 0.76), *p*<0.001; time on ART 12 to 18 months: IRR=0.48, 95% CI (0.40 to 0.58), *p*<0.001; and time on ART 18 to 24 months: IRR=0.42, 95% CI (0.34 to 0.51), *p*<0.001. The results suggested that both the GEE-logit model and the GEE-Poisson model are comparable when applied to our cohort data; therefore, we have chosen to maintain our original model in order to describe and interpret the effects of the covariates as ORs.

## Discussion

Approximately 32% of patients reported at least one adherence measurement <100% and 17% reported at least one measurement <95% during the 24-month follow-up time. We found that SubAdh to first-line ART in this cohort, defined as either <100% and <95%, was associated with mode of HIV exposure, ART regimen at initiation, time on ART and frequency of adherence measurement. IDUs were more likely to report being less adherent to their ART regimen, while those with homosexual HIV exposure were less likely to report lower adherence. Patients who were put on PI-containing regimens were more likely to be adherent compared to those who were given NNRTI-based regimens. The effects of increasing adherence with increasing time on ART were similar from both the main analyses and sensitivity analyses.

Clinical sites with an average adherence assessment of more than twice per patient per year had lower odds of SubAdh, indicating that the more frequently patients completed the VAS, the more adherent they became. We hypothesized that this could possibly be a reflection of the level of adherence counselling offered by clinical sites, that is, sites with a higher number of adherence measurement have more frequent patient contact, thus enabling low adherence to be identified early, and therefore allowing for more discussion and emphasis on the importance of adhering to the antiretroviral regimen. Patients’ level of engagement with healthcare providers has shown to be significantly associated with ART adherence in a multisite international study, which included sites from both resource-rich and resource-poor settings [18]. Therefore, the capacity for sites to provide appropriate levels of support and patient care can contribute significantly to patients’ adherence, which could lead to successful treatment outcomes.

The proportion of SubAdh was highest in the first six months of ART, and decreased thereafter. It is commonly known that loss to follow-up in the early stages of ART under resource-limited settings is high [3, 19]. Our data suggest that even after accounting for missing data in the sensitivity analyses, the adherence level maintained its improvement over time. This indicates that the significant effect of time was not entirely influenced by the losses to follow-up or missing values. Our findings are consistent with a cross-sectional multisite Nepalese study [4], where patients on shorter ART durations were more likely to be non-adherent than those who have been on for longer periods of time. This suggests that emphasis on adherence in the early months of ART should be a priority.

Our study did not find a significant association among the commonly known predictors of SubAdh such as age, sex and side effects [4, 5, 20–22]. However, the higher odds for IDUs found in this study are consistent with previous reports [21, 23, 24]. Homosexual HIV exposure was associated with better adherence. This is in contrast to previous findings [25] where rates of SubAdh in this group were higher. We hypothesize that this could be due to the increasing social support network for homosexual patients living with HIV, which may allow for better interaction and therefore encourage good adherence to ART [26, 27]. The use of PI-based regimens has been linked to poorer adherence outcome due to regimen intolerability in high-income settings [20, 28–30]. However, PI use in resource-limited settings is generally used for second-line ART [31]. Therefore, we believe that the positive effects of first-line PI users being more adherent might be related to the underlying site resourcing differences and unobserved socio-economic confounders [31]. To summarize, we believe that our findings of homosexual HIV exposure and PI-based regimens being associated with better adherence should be interpreted cautiously, as these findings are probably confounded by unobserved patient characteristics and other socio-economic risk factors known to affect adherence [32, 33].

The major limitations of our study included only having adherence data in the form of the self-reported VAS. No pharmacy pick-up or pill count data were available. It could be argued that using self-reported adherence may introduce social desirability bias where individuals may overestimate adherence levels [34, 35]. In particular, the more frequently adherence is assessed, the more likely individuals may adapt to reporting optimal adherence levels. However, a previous TASER-M analysis indicated that having self-reported SubAdh (<95%) was associated with virological failure [12]. This suggests that our adherence assessment questionnaire could be used as a tool to predict treatment responses and therefore was an appropriate measure of antiretroviral adherence in our clinical settings. We also did not obtain information on specific adherence management practices at the sites. However, we created a variable that summarized the median number of adherence assessments per patient per year for each site to attempt to explore the site-level effects. As this was an observational cohort, there were missing HIV outcome and follow-up data. Adverse events less than grade 3 were also not collected; therefore, it was not possible to determine the effect of all adverse events on adherence outcome. In addition, as already mentioned above, data on socio-economic factors were not available, which may have been significant confounders for adherence.

## Conclusions

In summary, suboptimal adherence was higher among IDUs and lower in patients with homosexual HIV exposure in our study cohort. The more frequently sites assessed their patients, the higher the adherence levels. Being on PI-based first-line regimens and longer times on ART was associated with improved adherence. A greater emphasis on more frequent adherence counselling immediately following ART initiation and through the first six months may be valuable in promoting treatment and programme retention.
